# Engineered collagen XVII-loaded dissolving microneedle patch for promoting hair regrowth in androgenic alopecia

**DOI:** 10.1093/rb/rbaf104

**Published:** 2025-11-09

**Authors:** Tao Ye, Chunna Wu, Yufei Fan, Huan Xia, Ziyi Li, Jingxian Deng, Ruxue Chang, Qihong Wu, Xun Tang, Tao Meng, Yifang Li, Yan Yang, Yadong Huang

**Affiliations:** Department of Cell Biology, Jinan University, Guangzhou 510632, China; Department of Cell Biology, Jinan University, Guangzhou 510632, China; Department of Cell Biology, Jinan University, Guangzhou 510632, China; Department of Cell Biology, Jinan University, Guangzhou 510632, China; Department of Cell Biology, Jinan University, Guangzhou 510632, China; Department of Cell Biology, Jinan University, Guangzhou 510632, China; Department of Cell Biology, Jinan University, Guangzhou 510632, China; State Key Laboratory of Bioactive Molecules and Druggability Assessment, Jinan University, Guangzhou 510632, China; Department of Cell Biology, Jinan University, Guangzhou 510632, China; Department of Cell Biology, Jinan University, Guangzhou 510632, China; Department of Cell Biology, Jinan University, Guangzhou 510632, China; Department of Cell Biology, Jinan University, Guangzhou 510632, China; National Engineering Research Center of Genetic Medicine, Jinan University, Guangzhou 510632, China; Guangdong Province Key Laboratory of Bioengineering Medicine, Jinan University, Guangzhou 510632, China; TYRAN Cosmetics Innovation Research Institute, Jinan University, Guangzhou 511447, China; Department of Cell Biology, Jinan University, Guangzhou 510632, China; State Key Laboratory of Bioactive Molecules and Druggability Assessment, Jinan University, Guangzhou 510632, China; National Engineering Research Center of Genetic Medicine, Jinan University, Guangzhou 510632, China; Guangdong Province Key Laboratory of Bioengineering Medicine, Jinan University, Guangzhou 510632, China

**Keywords:** recombinant human collagen XVII, microneedle patch, androgenic alopecia, hair regeneration

## Abstract

Androgenic alopecia (AGA), the most common form of progressive hair loss in both males and females, significantly impacts patients’ quality of life and confidence. Current therapies, such as minoxidil, are limited by poor patient compliance and low transdermal bioavailability, highlighting the need for more effective treatments. In this study, we identified collagen XVII (COL17) as a key player in AGA-like model pathogenesis, observing its significant downregulation in a testosterone-induced AGA-like mouse model. This reduction was accompanied by abnormal hair follicle morphology, decreased proliferation and impaired angiogenesis. To address this, we developed recombinant human COL17 fragment (800–1300 aa) (rhCOL17p) expressed and purified from *E. coli*, which demonstrated dose-dependent enhancement of dermal papilla cell adhesion, migration and proliferation *in vitro*. To overcome transdermal delivery challenges, we designed a dissolving microneedle (MN) patch using hyaluronic acid as a matrix. The rhCOL17p-MN achieved 96% skin penetration and sustained release of 96% within 28 h *in vitro*, with residual fluorescence detectable in mouse skin for up to 6 days. *In vivo*, the 4-mg/ml rhCOL17p-MN achieved a mean hair coverage of ∼97% by Day 14, which was statistically equivalent to the efficacy of 5% minoxidil, with increased follicle density, anagen-phase transition and CD31^+^ vascularization. Histological analysis revealed restored follicle structure and upregulated β-catenin^+^ and SRY-box gene 9 (SOX9^+^), indicating activation of stem cell and proliferative signaling pathways. The rhCOL17p-MN also demonstrated low hemolysis (<0.5%) and robust mechanical stability (≥0.2 N/needle), confirming its safety and feasibility. These findings establish COL17 downregulation as a critical mechanism in AGA and demonstrate that MN-delivered rhCOL17p promotes hair regeneration through multi-pathway regulation, offers preclinical evidence supporting its potential as a candidate strategy for further investigation in AGA-related research.

## Graphical Abstract

**Figure:**
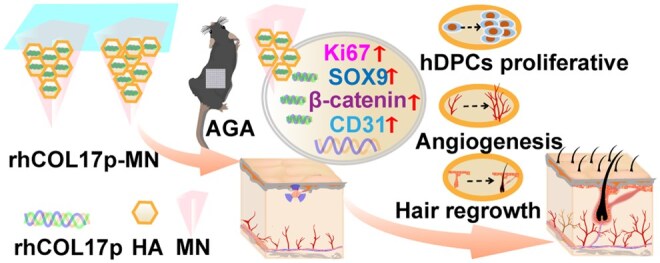

## Introduction

Hair loss, a globally prevalent degenerative skin condition, significantly impacts patients’ psychological well-being and quality of life [1, 2]. Among the various types of hair loss, androgenetic alopecia (AGA) accounts for over 90% of non-scarring alopecia cases [3]. The core pathological feature of AGA is the miniaturization of hair follicles, where terminal hairs progressively transform into vellus hairs [4]. This process is closely linked to androgen-induced dormancy of hair follicle stem cells (HFSCs) and dysregulation of signaling pathways [5, 6]. Despite its high prevalence, current therapeutic options, such as minoxidil and finasteride, are limited by low transdermal efficiency (<5%), systemic side effects and the need for daily administration, highlighting the urgent need for more effective and patient-friendly treatments [7–11].

Recent studies have shown that the dormancy-activation cycle of HFSCs is regulated by various transmembrane proteins and signaling pathways [12–15]. Among these, type XVII collagen (COL17), a core component of hemidesmosomes, has emerged as a critical molecular hub connecting the cytoskeleton to the ECM [16, 17]. COL17, a transmembrane collagen protein encoded by the *COL17A1* gene, plays a pivotal role in anchoring epidermal basal cells to the basement membrane through its trimeric structure [18, 19]. The intracellular domain of COL17 interacts with plectin and keratin intermediate filaments, while its extracellular domain engages with extracellular matrix (ECM) components, including integrin α6 and laminin, thereby maintaining the mechanical stability of the epidermis [20, 21]. Notably, COL17 is crucial for hair follicle regeneration by modulating the adhesion states and signal transduction of HFSCs [19, 22]. During anagen, high COL17 levels maintain the HFSC pool by promoting symmetric division, whereas COL17 deficiency tilts the balance toward asymmetric division, accelerates stem-cell exhaustion and—consistent with *COL17A1*-knockout mice—produces follicle miniaturization and premature hair loss, underscoring the essential role of COL17 in maintaining HFSC stemness [23–25]. However, in the context of AGA, the mechanisms by which downregulation of COL17 expression affects the quiescence-activation cycle of HFSCs remain to be fully elucidated.

In this study, we observed a significant downregulation of COL17 expression, which was accompanied by abnormal hair follicle morphology, reduced proliferation and impaired angiogenesis. To explore the therapeutic potential of COL17 in AGA, we designed a soluble recombinant human COL17p (rhCOL17p) through genetic engineering. Specifically, a 980-bp cDNA fragment encoding the key functional domain of the *COL17A1* gene was designed to produce a theoretical molecular weight 32.6 kDa soluble protein. This protein was expressed using an *E. coli* expression system and subsequently purified via nickel affinity chromatography. To enhance its delivery, we developed a MN hyaluronic acid (HA)/rhCOL17p delivery system, leveraging the transdermal capabilities of MN technology. In a testosterone-induced AGA-like mouse model, the rhCOL17p-MN patch effectively restored hair follicle morphology, promoted the transition to the anagen phase and enhanced angiogenesis. By integrating the regenerative properties of rhCOL17p with the advanced drug delivery capabilities of MN technology, this approach represents a promising strategy for improving hair regeneration and patient outcomes in AGA.

## Materials and methods

### Materials

Yeast extract (Cat No. LP0021T) and peptone (Cat No. LP0037B) were procured from Thermo Fisher Scientific (Shanghai, China). *Nco I* (Cat No. 1160S) and *Xho I* (Cat No. 1094S) restriction endonucleases were purchased from Takara Biotechnology Co., Ltd (Beijing, China). Isopropyl-β-D-Thiogalactoside (IPTG, Cat No. I6148) and Agar (Cat No. A6326) were purchased from Macklin Inc (Shanghai, China). Kanamycin Sulfate (Cat No. 367-93-1) was obtained from Sigma Aldrich Trading Co., Ltd (Shanghai, China). G-250 (Cat No. ST1119) was purchased from Beyotime Biotechnology Co., Ltd (Shanghai, China). HisTrap HP chromatography (Cat No. 17524802) column was purchased from Cytiva (Marlborough, USA). HA (Cat No. H823435, ∼50kDa) and pullulan (Cat No. 9057-02-7, ∼200kDa) were purchased from Macklin Biochemical Technology Co., Ltd (Shanghai, China). The DH5α Chemically Competent Cell (Cat No. CD201-01) and BL21(DE3) Chemically Competent Cell (Cat No. CD701-02) were purchased from TransGen Biotech Co., Ltd (Beijing, China). CCK8 (Cat No. HY-K0301) was purchased from MedChemExpress (Shanghai, China). Human dermal papilla cells (hDPCs, Cat No. IH1006) were purchased from Xinrun Biotechnology Co., Ltd (Wuxi, China).

### Establishment of male C57BL/6 mice AGA model

Six-week-old male C57BL/6 mice (serial number: 448827006839) were purchased from BesTest Co., Ltd (Zhuhai, China). All animal operations were performed according to the guidelines for the care and use of laboratory animals at Jinan University (Guangzhou, China) and approved by the Animal Ethics Committee of Jinan University. Firstly, all animals were housed in the animal room for 1 week and on Day 7, the dorsal skin was depilated to synchronize the hair cycle, and testosterone application was continued for a further 20 days [26]. Randomly divide the depilated mice into three groups (five mice were included in each group): the healthy group (mice without any treatment after depilation) and the androgen group (mice treated with 100 μL of 0.5% (w/v) testosterone solution applied evenly to the depilated dorsal skin once daily). Observations were conducted continuously for 20 days, starting from the first day after depilation (designated as Day 1). After successful modeling, the tissue was cut and fixed with 4% paraformaldehyde. Then, paraffin sections were made for H&E staining and immunohistochemical staining to analyze the changes in hair follicles and hCOL17 related to hair follicle growth in the skin tissue.

### Molecular design of hCOL17p

Bioinformatic analysis of hCOL17’s secondary and tertiary structures suggested that its C-terminal extracellular region (800 ∼ 1300 aa) likely harbors an independent functional peptide domain. We isolated this putative active fragment, named hCOL17p, and characterized its physicochemical and structural properties, laying a theoretical groundwork for recombinant rhCOL17p expression. The bioinformatics analysis tools were employed to predict and analyze the protein structure and functional characteristics of the functional fragment hCOL17p (Table 1).

**Table 1.** Main tools for bioinformatics analysis.

Protein structure prediction
SWISS-MODEL：http://swissmodel.expasy.org/； GOR4：https://npsa-prabi.ibcp.fr/cgi-bin/npsa_automat.pl?page=npsa_gor4. html NovoPro：https://www.novopro.cn/tools/ secondary-structure-prediction. html； Alphafold：https://alphafold.ebi.ac.uk/； Phyre2：https://www.sbg.bio.ic.ac.uk/∼phyre2/html/page.cgi? id=index； I-TASSER：https://zhanggroup.org/I-TASSER/output/S805114/

### Construction, expression and purification of rhCOL17p

#### Obtaining the hCOL17p gene

Human umbilical cord mesenchymal stem cells huc-MSC (1 × 10^5^ cells per well) were inoculated onto a 6-well culture plate. After culturing in DMEM medium (Cat No. 11965092, Thermo Fisher Scientific, Shanghai, China) containing 10% FBS (Cat No. FSP500, ExCell, Suzhou, China) for 72 h, huc-MSCs were washed twice with PBS. Total RNA was extracted from the cells using Trizol reagent (Cat No. 15596026CN, Invitrogen, USA), followed by Prime Script™ RT kit (TaKaRa Bio, Co., Ltd, Dalian, China), was reverse transcribed into cDNA using reverse transcriptase. The hCOL17p subclonal sequence was amplified from the cDNA template by PCR and identified by Beijing Qingke Biotechnology Co., Ltd. The sequences of primers were as follows: hCOL17p-F (5′-TCC ACA GCT TG G ATG GGC CCA CCA GGC CCA CCA GGA T-3′), hCOL17p-R (5′-TCG AGT GCG CGG CCT CAG CGC GTA GTA TGT AAG TAA G-3′).

#### Construction of recombinant plasmid pET28a-hCOL17p

The subclone hCOL17p sequence was amplified from the cDNA template by PCR, and the PCR product and pET28a vector were digested with *Nco I* and *Xho I* restriction endonucleases at 37°C. The hCOL17p fragment and pET28a vector fragment were recovered from the kit, and the two fragments were ligated using T4 ligase under overnight conditions at 4°C to construct the recombinant plasmid pET28a-hCOL17p. The recombinant plasmid was transferred to the competent DH5α state after 90 s of heat shock at 42°C. The next day, monoclonal bacteria were selected from the plate for colony PCR validation of positive transformants.

#### Expression of recombinant plasmid pET28a-hCOL17p in host

The recombinant plasmid pET28a-hCOL17p was subjected to heat shock at 42°C for 90 s and transferred to host competent states BL21(DE3). Positive monoclonal bacteria were selected and inoculated into a 5 mL LB medium containing 50 μg/mL kanamycin at 37°C and 200 rpm overnight. The next day, the bacterial solution was transferred to 50 mL LB medium at a ratio of 1% (v/v) and shaken for 3 h. The OD_600_ of the bacterial solution reached 0.8. One millimolar of IPTG inducer was added to induce expression at 37°C for 3 h. After centrifugation at 12 000 rpm at 4°C for 5 min, the supernatant was discarded, and the bacterial cells were resuspended in 5% SDS (Cat No. 28364, Thermo Fisher Scientific, Shanghai, China). After boiling the bacterial cells for 10 min and centrifuging at 12 000 rpm at 4°C for 5 min, the supernatant collected was analyzed for the expression level of rhCOL17p using SDS–PAGE.

#### Purification and western-blotting analysis of rhCOL17p

The rhCOL17p protein was purified by affinity chromatography on Ni Sepharose™ 6 Fast Flow (Cat No. 17531803, Cytiva, Marlborough, USA) column combined with gel filtration of Sephadex G-25. It was combined with Orbitrap Fusion Lumos Tribrid (Thermo Fisher Scientific, USA). The western blotting is used for protein purity analysis of rhCOL17p.

### hDPCs cell identification

In order to further verify whether rhCOL17p affects the formation of hair follicles during hair growth, this study measured the expression levels of K15 and K19 related to hair follicle proliferation in mice skin using immunohistochemistry [27, 28]. We obtained hDPCs (Cat No. IH1006) from Xinrun Biotechnology Co., Ltd (Wuxi, China) and detected their specific marker genes *ALP, SOX2, α-SMA, Nestin* and *Versican* using RT-PCR [29–33]. We also measured the particular markers SOX2, α-SMA, K15 and K19 using immunofluorescence. After identification, the cells were passaged in a DMEM medium containing 10% FBS, and 7–10 passages of hDPCs were selected as the validation for all subsequent experiments (Table 2).

**Table 2.** Primer sequences used for RT-PCR.

Gene	Primer sequences
*Nestin*	F：5′-CAGCGTTGGAACAGAGGTTGGAG-3′
	R：5′-AAGGGTAGCAGGCAAGGGTGAG-3′
*α-SMA*	F：5′-CTCTGGACGCACAACTGGCATC-3′
	R：5′-CCCATCAGGCAACTCGTAACTCTTC-3′
*Versican*	F：5′-ACGGCTTTGACCAGTGCGATTAC-3′
	R：5′-AACACAAGTGGCTCCATTACGACAG-3′
*SOX2*	F：5′-CAACATGATGGAGACGGAGCTGAAG-3′
	R：5′-CCGCTTAGCCTCGTCGATGAAC-3′
*ALP*	F：5′-GTTCGACGACGCCATTGAGAGG-3′
	R：5′-CACTGCTGACTGCTGCCGATAC-3′
*GAPDH*	F：5′-GGTGAAGGTCGGTGTGAACG-3′
	R：5′-CTCGCTCCTGGAAGATGGTG-3′

### rhCOL17p in hDPCs viability assay

hDPCs were transferred into DMEM containing 10% FBS and cultured at 37°C with 5% CO_2_. CCK8 assay was used to evaluate the proliferation activity of hDPCs at different protein concentrations. There are two groups, the control group (without any treatment) and the treatment group rhCOL17p (i.e. 1.56, 3.13, 6.25, 12.50 and 25.00 μg/mL). Each concentration of rhCOL17p was tested in three replicate wells, and the experiment was independently repeated 3 times, and the absorbance values of different groups are measured at a wavelength of 450 nm [34].


$$\mathrm{Cell}\;\mathrm{proliferation}\;\mathrm{rate}\;\mathrm{(\%)}=\frac{\mathrm{OD}\;\mathrm{treatment}\;\mathrm{group}-\mathrm{OD}\;\mathrm{control}\;\mathrm{group}}{\mathrm{OD}\;\mathrm{control}\;\mathrm{group}}\times 100\%$$

Cell adhesion activity was measured using the crystal violet method [34]. hDPCs were seeded on a 96-well plate and inoculated into the control group (without any treatment) and treatment group rhCOL17p (i.e. 1.56, 3.13, 6.25, 12.50 and 25.00 μg/mL), respectively. The cells were cultured at 37°C with 5% CO_2_ for 4 h, washed 3 times with 1 M PBS, shaken dry and then fixed with 200 μL of 4% paraformaldehyde for 20 min. The cells were stained with 1% crystal violet for 20 min. Each concentration of rhCOL17p was tested in three replicate wells, and the experiment was independently repeated 3 times. The adhesion of cells in different groups and protein treatment concentrations was observed under a microscope, and the cell adhesion effect was calculated using Image J software.

An *in vitro* scratch healing model was used to evaluate the induction of cell migration using recombinant collagen rhCOL17p. hDPCs in a 12-well plate, add DMEM medium containing 10% FBS, and culture at 37°C with 5% CO_2_. Before scratching, hDPCs were cultured to over 95% in each well, and the adherent cells were scratched using a 1 mL sterile pipette tip. After scratching, the cells were washed with PBS to remove scratched cell debris. Then, the control group (without any treatment) and treatment group rhCOL17p (i.e. 1.56, 3.13, 6.25, 12.50 and 25.00 μg/mL) were added to the cells. Each concentration of rhCOL17p was tested in three replicate wells, and the experiment was independently repeated 3 times. Using an inverted microscope, observe the cell migration at scratch sites at 0, 24 and 48 h, and analyze the images of the scratch area using Image J software. Measure the distance of cell migration and calculate the cell migration rate [35].


$$\mathrm{Cell}\;\mathrm{migration}\;\mathrm{rate}\;(\%)=\frac{\mathrm{Scratch}\;\mathrm{width}\;\mathrm{of}\;\mathrm{treatment}\;\mathrm{groups}-\mathrm{Control}\;\mathrm{group}}{\mathrm{Scratch}\;\mathrm{width}\;\mathrm{of}\;\mathrm{control}\;\mathrm{group}\;}\times 100\%$$

### Preparation and characterization of MNs patches

Weigh different weights of HA (Cat No. H823435, Macklin Biochemical Technology Co., Ltd, Shanghai, China) and dissolve them in ddH_2_O at four concentrations of 10%, 20%, 30% and 40% (w/v). Stir evenly at room temperature to prepare HA solutions. Four different concentrations of HA solutions were used to prepare MN patches, and the preparation process was completed under normal temperature and humidity conditions. Firstly, pour 100 μl of HA solution into a polydimethylsiloxane (PDMS, Cat No. D849784, Macklin Biochemical Technology Co., Ltd, Shanghai, China) master mold (with a base diameter of 350 μm, height of 750 μm, needle tip of 16 μm, size of 14.5 mm × 14.5 mm, array of 15 mm × 15 mm and tip spacing of 720 μm). Then, place it in a vacuum pump with a pressure of 0.1 MPa for 5 min, centrifuge the PDMS mold at 4000 rpm for 5 min and fill the mold cavity with the suspension. Finally, add 100 μl of pullulan sugar solution to the PDMS mold’s upper layer to form the patch’s backing layer. Dry at 20°C for 8 h, becoming an MN patch after demolding. The characterization of the MN patch is observed by an optical microscope.

### Preparation and characterization of rhCOL17p-MN patch

Firstly, 3 g of HA powder was thoroughly mixed with rhCOL17p solutions of 1, 2 and 4 mg/mL to prepare a HA/rhCOL17p matrix solution containing 30% HA. Adding 30% HA can provide sufficient mechanical strength and good morphology for MNs and enhance their solubility in the skin. Secondly, 100 μL of HA/rhCOL17p matrix solution was filled onto a PDMS mold, and after vacuum filtration at 0.1 MPa for 5 min, the PDMS mold was centrifuged at 4000 rpm for 5 min to fill the cavity of the mold with the suspension. Finally, 100 μl of pullulan solution was added to the PDMS mold’s upper layer to form the patch’s backing layer. After drying at 20°C for 8 h, the rhCOL17p-MN patch was de-molded and stored at −20°C for future use. The shape and surface morphology of the rhCOL17p-MN patch were observed by SEM and optical microscope.

### rhCOL17p-MN patch hemolysis test

Two millimeters of whole blood from the tail vein of SD rats were collected and placed in an EP tube containing heparin. Centrifuge the collected blood at 10 000 rpm for 5 min, discard the supernatant and rinse the collected red blood cells with 2 mL of normal saline. Then, the red blood cells were centrifuged at 3000 rpm for 5 min and washed thrice. Finally, washed red blood cells were re-suspended in physiological saline to a 2% (v/v) suspension (equivalent to 8 × 10^9^ cell/mL). Then, different concentrations of rhCOL17p-MN patches were dissolved in physiological saline containing red blood cells as the experimental group, and deionized water and physiological saline were used as the positive and negative control groups. After co-culturing at 37°C for 1 h, the supernatant was centrifuged at 13 000 rpm for 10 min and placed in a 96-well plate. The absorbance at a wavelength of 540 nm was measured using a microplate reader. The formula for calculating the hemolysis rate (%) is as follows [36]:


$$\mathrm{Hemolysis}\;\mathrm{rate}\;(\%)=\frac{\mathrm{Ae}-\;\mathrm{An}}{\mathrm{Ap}-\mathrm{An}\;}\times 100\%$$

Ae is the absorbance value of the experimental group, An is the absorbance value of the negative control group and Ap is the absorbance value of the positive control group.

### Mechanical properties and penetration effect of rhCOL17p-MN patch

#### Mechanical performance

At a displacement force testing station, the rhCOL17p-MN patch was measured using an electric universal testing machine (ELF 3200, Bose, USA). In short, connect the rhCOL17p-MN patch to a rigid platform with the needle surface facing upward, and use the MN/HA patch as the control group. The instrument testing station moves the sensor probe from the vertical direction toward the tip of the MN patch at a speed of 1.8 mm/min, with an initial distance of 2.0 cm between the sensor and the MN patch. When the sensor first contacts the tip of the MN patch, it begins to record displacement and force measurements and continues until the sensor moves 0.8 mm from the tip of the MN patch toward the backing of the patch. The instrument will issue a warning indicating that the displacement has reached its maximum and the force on the MN patch has reached its limit. Measure the voltage resistance of the rhCOL17p-MN patch through displacement and force measurements. Simultaneously pierce the rhCOL17p-MN patch through eight layers of sealing film, and calculate the puncture rate of the rhCOL17p-MN patch on the sealing film.

#### Delivery efficiency

In order to evaluate the delivery and penetration efficiency of the rhCOL17p-MN patch on the skin, rhCOL17p was mixed with nonfluorescent dye methylene blue to prepare the rhCOL17p-MN patch according to the preparation method of rhCOL17p-MN patch. Place the needle tip tightly against the skin surface of the shaved mice back hair (2 cm × 3 cm), press with the thumb using ∼0.5 Newtons of force for 120 s, observe the dye remaining on the skin surface in the MN patch and calculate the delivery efficiency by dividing the dye delivered in the skin by the amount of dye in the MN patch before insertion.

### Healing effect of rhCOL17p-MN patch skin implantation

In order to evaluate the healing effect of the rhCOL17p-MN patch implanted into the skin, the tip of the rhCOL17p-MN patch was pressed tightly against the shaved back hair (2 cm × 3 cm) of the mice skin, press with the thumb using ∼0.5 Newtons of force for 120 s. After staying on the skin for different periods (i.e. 0, 15 and 30 min), the patch backing was removed, and the healing effect of the mice skin at different periods was observed under an optical microscope.

### *In vitro* dissolution time of blank (HA/pullulan) MNs

Dissolution kinetics of blank MN (30% w/v HA, 20% w/v pullulan backing) were determined at 37°C under sink conditions (PBS, pH 7.4, 100 rpm). Needle height was monitored by optical microscopy every 1 min until complete loss of pyramid morphology.

### Investigation of transdermal absorption of rhCOL17p in skin

Franz diffusion cells were employed to evaluate the transdermal delivery efficiency of rhCOL17p-MN *in vitro*. Fresh porcine skin was punctured with crosslinked MNs loaded with different concentrations of FITC-rhCOL17p-MN (e.g. 1, 2 and 4 mg/mL), then clamped between the donor and receptor chambers of the diffusion cell with the dermal side facing the receptor solution. The donor chamber was covered with aluminum foil to protect from light and prevent moisture loss. The receptor chamber was filled with 8 mL of PBS (pH = 7.8), and the sampling arm of the diffusion cell was sealed. A magnetic stir bar was placed inside the diffusion cell, and the system was continuously stirred at 600 rpm and 37°C. At predetermined time points, 200 μL of dialysate was collected from the diffusion cell, and an equal volume of PBS was replenished. FITC-loaded MN served as the control group. The fluorescence intensity of the sample solutions was analyzed using a multifunctional microplate reader, and the readings were fitted to a standard curve to calculate the concentrations of FITC-MN and FITC-rhCOL17p-MN. This allowed for the observation of the transdermal absorption of FITC-rhCOL17p-MN in the skin at different concentrations.

### Release and degradation of rhCOL17p-MN patch in animal tissues

In order to evaluate the release and degradation of rhCOL17p in the skin of rhCOL17p-MN patches, rhCOL17p was crosslinked with fluorescent dye FITC. The crosslinked protein FITC-rhCOL17p was prepared according to the preparation method of rhCOL17p-MN patches. The fluorescent FITC-rhCOL17p-MN patch was applied to simulate drug release kinetics. Before applying the FITC-rhCOL17p-MN patch, after shaving the hair on the back of the mice (2 cm × 3 cm), the fluorescent FITC-rhCOL17p-MN patch was inserted into the shaved area on the back of the mice. After pressing for 120 s, the MN remained on the skin for 20 min, indicating that FITC-rhCOL17p-MN could completely dissolve in the skin. In order to investigate the release of FITC-rhCOL17p, a small animal *in vivo* imaging device was used to observe the changes in fluorescence intensity of FITC-rhCOL17p-MN patches in mice skin at different time periods (i.e. 0, 3, 6 and 9 days). The fluorescence intensity of the implanted fluorescent patch on the back skin of mice was quantitatively analyzed using Image J software.

### Treatment of AGA animal model with rhCOL17p-MN patch

Six-week-old C57BL/6 males were housed in standard animal cages, fed a basic diet and kept in a light/dark cycle for 12 h. The room temperature was maintained at 23 ± 2°C, and the humidity was appropriate. After being fed adaptively for 1 week, the mice were anesthetized with an intraperitoneal injection of 1% sodium pentobarbital. Then, the hair on the back of the mice was shaved off using an electric hair clipper and depilated with hair removal cream. Mice were randomly divided into six groups, with five mice per group, namely the model group, blank MN group, rhCOL17p-MN (1 mg/mL) group, rhCOL17p-MN (2 mg/mL) group, rhCOL17p-MN (4 mg/mL) group and 5% minoxidil group. All groups of mice were locally coated with a 0.5% testosterone (50% ethanol solution) solution (10 mg/kg) in the depilation area to establish an AGA mice model. This was done once a day for 14 consecutive days, and the hair regeneration diameter, hair coverage rate and hair density in the depilation area of the mice were observed. After euthanizing mice, the skin tissue of the patch site was cut and fixed with 4% paraformaldehyde, embedded in paraffin and made into 10 μm thick sections. The hair follicle regeneration and dermal thickness were measured by H&E staining, and the expression levels of CD31, SOX9, β-catenin and Ki67 in the skin tissue were evaluated by immunohistochemical staining. All animal operations were performed according to the guidelines for the care and use of laboratory animals at Jinan University (Guangzhou, China) and approved by the Animal Ethics Committee of Jinan University (approval number: IACUC issue No.: 20210629-06, approval date: 29 June, 2021).

### Power calculation

A prospective power analysis (G*Power 3.1, two-tailed *t*-test, α = 0.05, power = 0.80, effect size = 1.2) indicated *n* = 5 animals per group as the minimum required to detect a 20% difference in hair-coverage percentage. This effect size was based on a pilot study (*n* = 3) showing 18% mean difference between testosterone-only and 4 mg/mL rhCOL17p-MN.

### Safety evaluation of rhCOL17p-MN patch

C57BL/6J mice were depilated and randomly divided into five groups: blank MN, rhCOL17p-MN (1, 2, 4 mg/mL) and control (no treatment). Patches were administered daily for 28 days. Serum levels of ALT, AST, BUN, UA and CRE were measured to assess liver and kidney function. Major organs (heart, spleen, liver, lungs, kidneys) were collected for H&E staining to evaluate tissue morphology and safety.

### Acquisition protocol

All dorsal photographs were taken with a Canon EOS 90D fixed 35 cm above the mouse back. Camera settings: manual mode, ISO 100, 1/125 s, f/8, 5500 K custom white-balance. Illumination: four 65 W 5500 K LED panels at 45° angles; intensity verified with a Sekonic L-308X light-meter (± 5%). All images were captured under identical lighting conditions (D65 standard daylight, 5000K color temperature) during the experiment. For post-processing, we unified the white balance using the ‘neutral gray’ tool on the non-hair, non-pigmented skin area of each image, eliminating lighting-induced color variations between panels.

### Hair-growth photography and color calibration

To ensure objective and reproducible recording of dorsal hair regrowth, all mice were photographed under a standardized imaging booth. A Canon EOS 90D camera (35 mm fixed focal length) was mounted 35 cm above the mouse back and operated in manual mode (ISO 100, 1/125 s, f/8). Custom white-balance was set to 5500 K using an 18% neutral-gray card (X-Rite ColorChecker). Illumination consisted of four 65 W 5500 K LED panels positioned at 45° angles to the vertical axis; incident intensity was verified with a Sekonic L-308X light-meter (± 5% tolerance). The gray card was placed beside the animal in every frame and retained in the final image for *post hoc* verification. Images were white-balanced to the gray-card reference using X-Rite ColorChecker Passport software and exported to sRGB IEC61966-2-1 color space; no further tonal or color adjustments were applied. All images were captured under these identical settings.

### Statistical analysis

All data were obtained from three independent experiments performed in triplicate and evaluated by one-way ANOVA analysis with GraphPad Prism. The results were displayed as means±the standard errors (SE). Differences were considered statistically significant if *P *< 0.05 (*P < 0.05, **P < 0.01, ***P < 0.001 and ****P < 0.0001).

## Results

### COL17 downregulation in a testosterone-induced AGA-like mouse model

**Figure 1.**
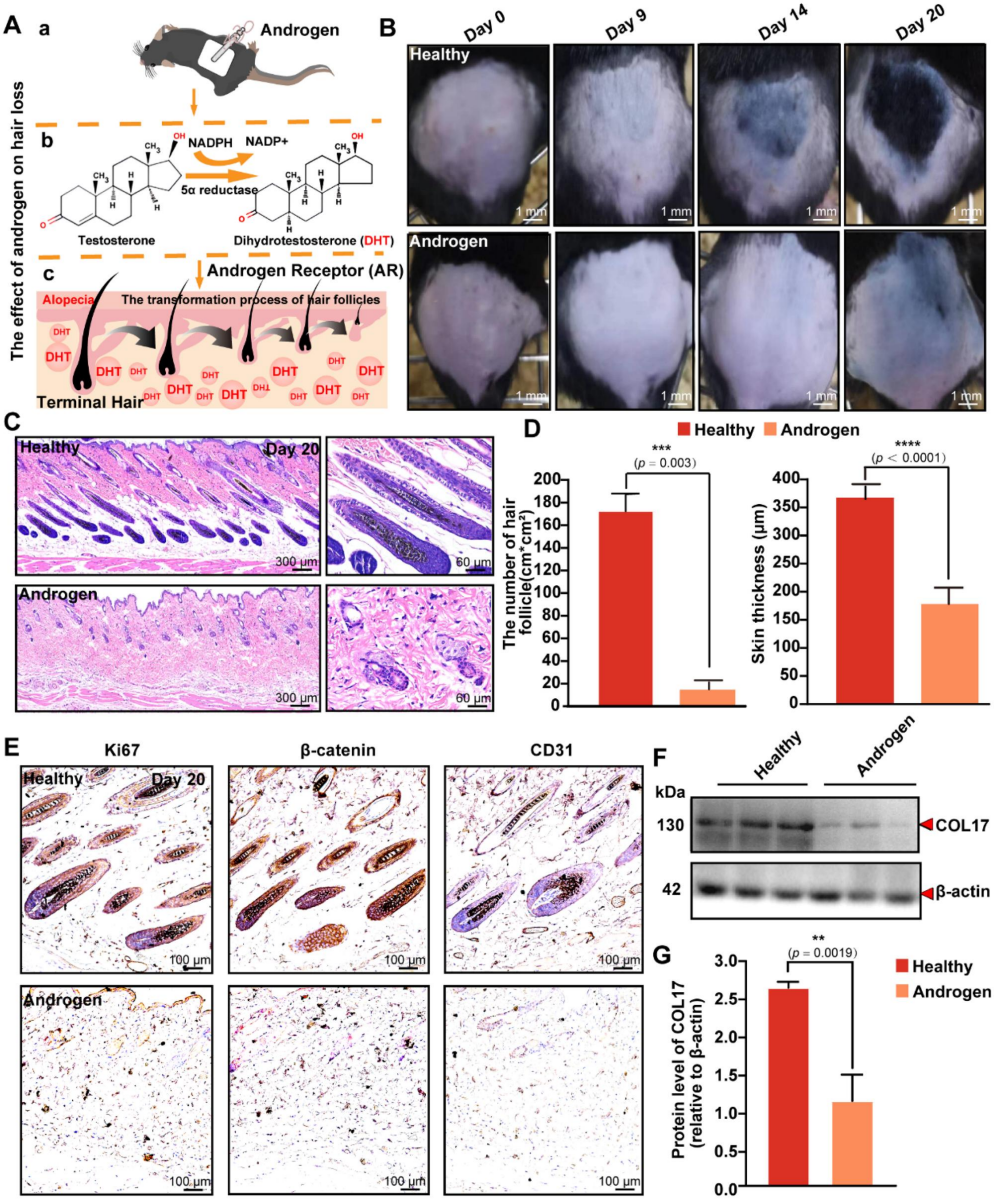
Establishment of AGA-like mouse model. (**A**) Schematic diagram of AGA model. (**B**) Representative optical images of mice treated with testosterone. (**C**) H&E staining on Day 20 after AGA model. (**D**) Hair follicles (e.g. follicles/cm²) and skin thickness on Day 20 after AGA model. (**E**) Representative immunohistochemical staining images of Ki67, β-catenin and CD31 indicating newborn hair follicles on the 20th day. (**F**) Expression level of COL17 was examined for COLXVII antibody by western blotting between healthy and androgen. (**G**) Protein level COL17 by Image J. Each bar represents the mean±SD (*n* = 3).*P < 0.05, **P < 0.01, ***P < 0.001 and ****P < 0.0001.

In this study, we established an AGA-like mouse model to investigate the pathological mechanisms and therapeutic interventions for hair loss (Figure 1A). To induce androgenetic alopecia (AGA)-like phenotypes, we topically applied 0.5% (w/v) testosterone to the dorsal skin of mice once daily. After nine consecutive days of treatment, testosterone-treated mice showed no melanin production, indicating delayed hair-follicle regeneration, whereas control mice had already begun to produce melanin in certain areas. By the 14th day, robust hair growth was observed in controls, but testosterone-treated mice displayed no significant melanin aggregation. By the 20th day, the hair coverage rate in controls exceeded 80%, compared to less than 10% in the testosterone group (Figure 1B). Hematoxylin and eosin (H&E) staining results showed that the hair follicles in the skin tissue of the control group mice had normal morphology and growth, with uniform arrangement and distribution, and varying sizes, indicating that the hair follicles were in the proliferative and division stage (Figure 1C). However, the hair follicles in the skin tissues of the testosterone-treatment mice exhibited abnormal morphology and growth, characterized by hair follicle shrinkage and degeneration, and significant tissue proliferation below the hair follicle site (Figure 1D). Further analysis showed that the expression levels of key markers, including Ki67 (cell proliferation), CD31 (endothelial cells) and β-catenin (hair follicle development and maintenance), were significantly downregulated in the testosterone-treated group compared to the controls (Figure 1E). These findings collectively indicated that testosterone treatment disrupted hair follicle integrity, impaired proliferation, and inhibited hair follicle development and maintenance, further supporting the successful induction of AGA-like phenotypes in this model. Additionally, we found that the relative expression level of COL17 in the depilated area tissue of the control group mice was significantly higher than that in the testosterone groups (Figure 1F and G). Its low expression was positively correlated with the occurrence of AGA. This suggested that COL17 may play a critical role in the pathogenesis of AGA.

### Molecular design of the functional fragment hCOL17p

**Figure 2.**
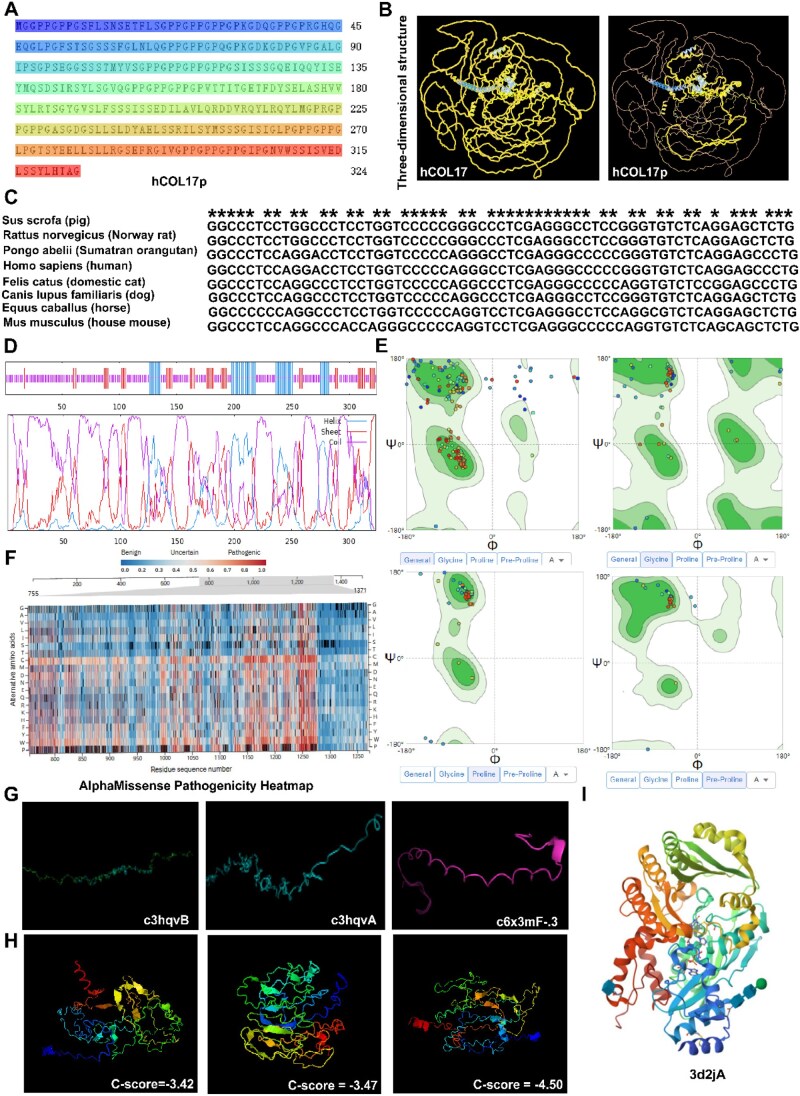
Protein structure analysis of hCOL17p. (**A**) The amino acid sequence of hCOL17p protein. (**B**) 3D structural comparison of full-length protein hCOL17 versus recombinant protein hCOL17p. (**C**) Conserved active site retention in multiple sequence alignment from different species. (**D**) Secondary structure analysis of hCOL17p polypeptide chain. (**E**) Ramachandran plot of hCOL17p. (**F**) AlphaMissense pathogenicity heatmap. (**G**) Folded regions of the advanced structure of hCOL17p. (**H**) Structural cluster of hCOL17p. (**I**) The active site of protein structure of hCOL17p.

Given the established correlation between COL17 downregulation and AGA progression, we aimed to develop hCOL17p as a potential therapeutic intervention. To identify a bioactive functional fragment, we extracted the 800–1300 amino acid sequence from the extracellular domain of COL17, designated as hCOL17p (Figure 2A). Bioinformatics analysis revealed its key features (Supplementary Table S1): ProtParam (ExPASy) predicted theoretical molecular weights of 150.4 kDa for full-length hCOL17 and 32.6 kDa for hCOL17p, with isoelectric points (pI) of 8.89 and 4.58, respectively, and hydrophilic GRAVY indices (−0.573 and −0.416) supporting its suitability for recombinant expression. SWISS-MODEL homology modeling and Ramachandran plots (Figure 2E) indicated a reasonable conformational distribution, while AlphaMissense pathogenicity scoring (average pLDDT 40.38) detected no pathogenic regions, with benign regions concentrated at the C-terminus (Figure 2F). Phyre2 folding analysis revealed high freedom degrees (88.1–97.6%) in key regions (Figure 2G), and I-TASSER generated five structural clusters, with models scoring −3.42 to −4.50 exhibiting optimal quality (Figure 2H). Domain analysis highlighted the LisH domain (127–139 aa) for Set3 complex interactions and the 173–194 aa domain for regulating cell proliferation and migration, often in synergy with WD-40 and other functional domains (Supplementary Table S2).

### Recombinant expression and construction of hCOL17p

**Figure 3.**
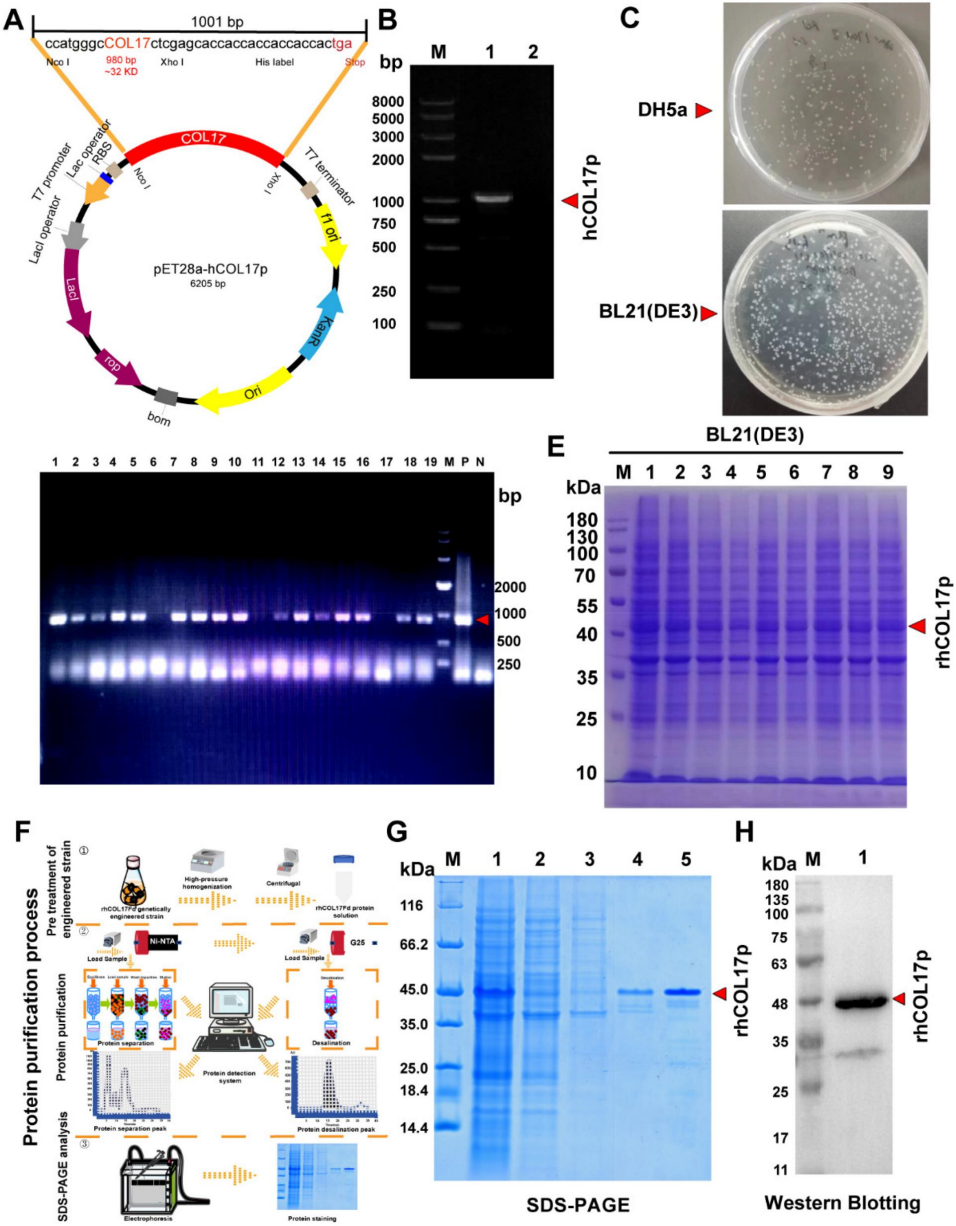
Expression of the hCOL17p gene. (**A**) Recombinant construct map of the expression vector pET28a-hCOL17p. The synthetic gene, the pET28a, Escherichia coli expression vector and the resulting pET28a-hCOL17p plasmid are shown. In the schematic plasmid map, the hCOL17p gene and the location of the *nco I* and *XhoI* restriction sites are represented. Other genetic elements, as the T7 promoter, the Lac operator, the *lacI* gene, the *pBR322* replication origin and the kanamycin resistance gene, were omitted for clarity. (**B**) hCOL17p gene by PCR identification. (**C**) *E. coli/*DH5α and *E. coli/*BL21 (DE3) competent cells were transformed with the pET28ahCOL17p plasmid. (**D**) Nucleic acid gel electrophoresis identification of positive transformants after colony PCR. M: 5000 bp DNA marker. (**E**) SDS–PAGE detection of rhCOL17p expression in different expression strains. M: 180 kDa protein marker. (**F**) Purification diagram of rhCOL17p protein. (**G**) Protein purity assessment of rhCOL17p by SDS–PAGE. M: Protein marker (14.4 ∼ 116 kDa); lane 1: centrifuged sample after cell homogenization; lane 2: flow-through sample; lane 3: wash A; lane 4: wash B; lane 5: elution A. (**H**) Western blotting identification of rhCOL17p (M: protein marker; lane 1: rhCOL17p).

To produce the soluble recombinant protein, a 980-bp cDNA fragment encoding the critical functional domain of hCOL17p was cloned into the pET28a vector using *Nco I/Xho I* restriction sites, incorporating a C-terminal 6×His tag for purification (Figure 3A and B). The resulting 6205 bp pET28a-hCOL17p plasmid was transformed into DH5α and BL21(DE3) competent cells (Figure 3C), and positive transformants were identified by PCR (Figure 3D). The calculated molecular mass of the hCOL17p polypeptide (800–1300 amino acids) is 32.6 kDa. However, the apparent molecular mass of the purified His-tagged construct, as determined by SDS–PAGE, is ∼45 kDa (Figure 3E). A two-step purification strategy was employed: initial nickel affinity chromatography (Ni Sepharose 6 Fast Flow) achieved 85% purity (Figure 3F), followed by gel filtration (Sephadex G-25) for further refinement. The identity of the purified protein was rigorously validated through Coomassie staining (Figure 3G), anti-His western blot (Figure 3H). These results collectively confirmed the successful production of rhCOL17p for functional studies.

### rhCOL17p promotes hDPCs proliferation, adhesion and migration

**Figure 4.**
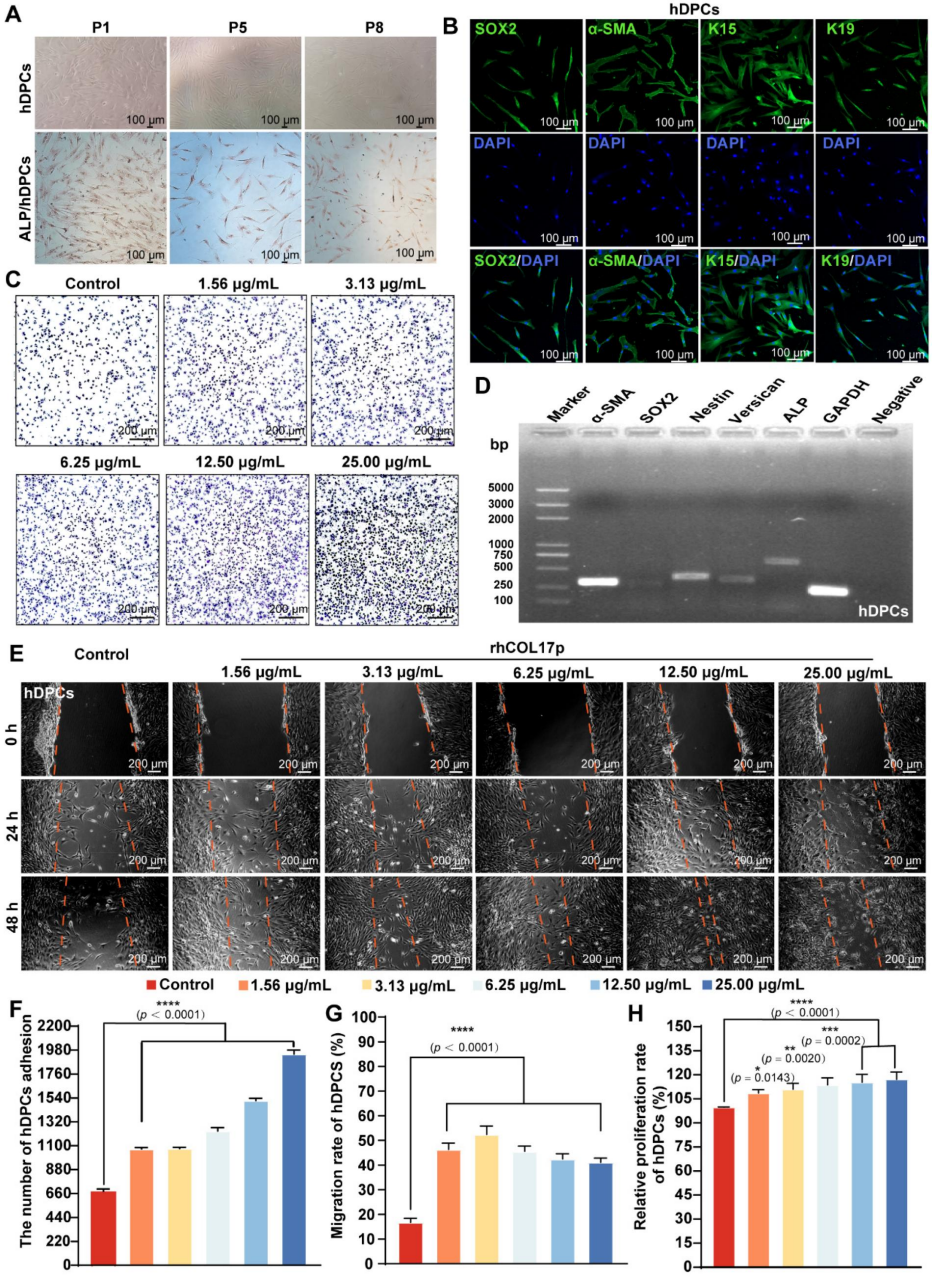
Culture, identification and biological activity of hDPCs. (**A**) Different generations (P1, P5, P8) of hDPCs cultivation by ALP staining. (**B**) Representative immunofluorescence staining images of SOX2, α-SMA, cytokeratin 15 and 19 indicating newborn hair follicles. (**C, F**) The adhesion of hDPCs cultured on rhCOL17p. Scale bars, 200 μm. (**D**) RT-PCR was used to detect the specific marker genes *ALP, SOX2, α-SMA, nestin* and *versican* in hDPCs. (**E, G**) The migration characteristics of hDPCs on rhCOL17p were examined by the scratch wound assay in 24 and 48 h. Scale bars, 200 μm. (**H**) The proliferation assay of hDPCs on rhCOL17p. Each bar represents the mean±SD (*n* = 3). compared with the control group, *P < 0.05, **P < 0.01, ***P < 0.001 and ****P < 0.0001.

To investigate the promotive effects of rhCOL17p *in vitro*, we employed a series of experimental approaches to evaluate its impact on key cellular functions, including adhesion, migration, and proliferation. Initial characterization of hDPCs confirmed their identity, with marker gene expression showing a 100% positivity rate (Figure 4A, B, and D). When cultured on rhCOL17p-coated substrates, hDPCs exhibited dose-dependent enhancement in cell adhesion, as quantified by crystal violet staining, with maximal efficacy at 25 μg/mL. The *in vitro* scratch healing test was used to assess the migration characteristics of hDPCs at concentrations of 1.56, 3.13, 6.25, 12.50 and 25.00 μg/mL of rhCOL17p. The cell migration assay demonstrated that treatment with varying concentrations of rhCOL17p for 24 and 48 h significantly enhanced cell migration in a dose-dependent manner (Figure 4C and F). Notably, at a concentration of 25 μg/ml, the migration rate reached approximately (44.46 ± 4.30)% compared to (16.79 ± 1.60)% in the control group (Figure 4E and G). Finally, the CCK8 assay evaluated the proliferation effect of rhCOL17p on hDPCs and showed that, compared to the control group, rhCOL17p significantly promoted hDPCs proliferation without a clear dose-dependent trend (Figure 4H). These findings collectively suggested that rhCOL17p has significant effects on enhancing cell adhesion, migration and proliferation in hDPCs.

### Fabrication and characterization of the rhCOL17p-loaded dissolving MN patch (rhCOL17p-MN)

**Figure 5.**
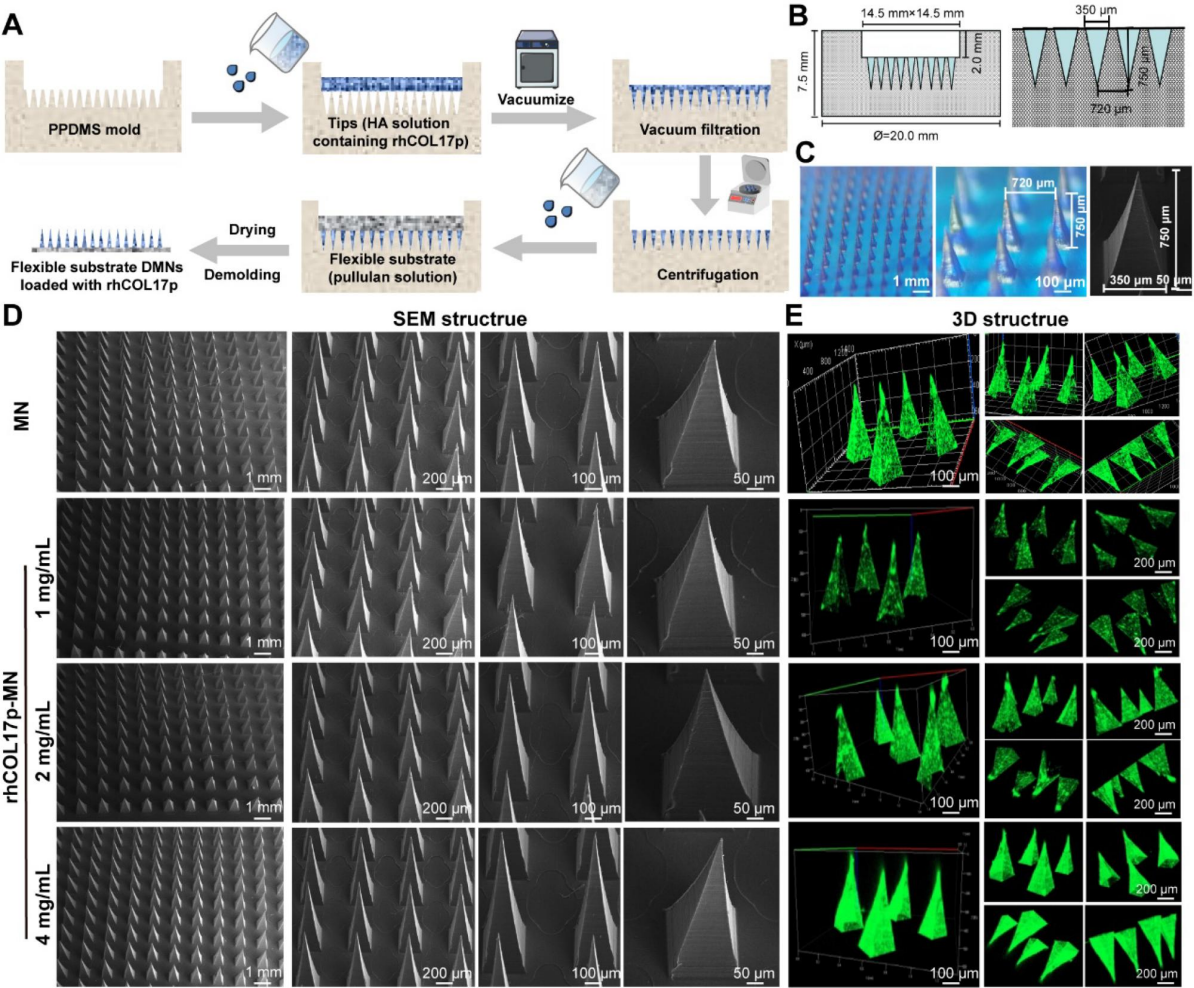
Fabrication and characterization of rhCOL17p-MN patch. (**A**) Fabrication process of MN patches by using PDMS molds. (**B**) Specifications of PDMS microneedle mold. (**C**) Representative matrix of microneedles (left), specification parameters of microneedle under optical microscope (middle), parameters of a single microneedle under SEM (right). (**D**) SEM images of microneedle patches at concentrations of 0, 1, 2, and 4 mg/ml of rhCOL17p. (**E**) 3D structure of microneedle patches at concentrations of 0, 1, 2, and 4 mg/ml of rhCOL17p.

To fabricate the dissolving MN patch loaded with rhCOL17p, a two-step casting procedure was implemented (Figure 5A). HA, a safe substance approved by the FDA for use as a dermal filler, was utilized as the matrix for the dissolvable needles [37–39]. These needles can remain within the skin, dissolve rapidly and ultimately degrade [40]. In the initial step, mixtures of rhCOL17p at concentrations of 1, 2 and 4 mg/ml, in a 7:3 ratio with the HA solution, were filled into the PDMS mold to form the needle-tips (Figure 5B). Subsequently, pullulan sugar solution was coated into the mold to form the pedestals. The resulting rhCOL17p-MNs consisted of 15 × 15 needle arrays. Each needle was pyramid-shaped, with a height of 750 μm, a width of 350 μm and an interval of 720 μm (Figure 5C). SEM observation verified that the needles in all groups were evenly arranged, were pyramid-shaped with a base length of 350 μm and a height of 750 μm, and had sharp tips without burrs (Figure 5D).

For observing the distribution of rhCOL17p within the MNs, rhCOL17p was labeled with fluorescein isothiocyanate (FITC) and then loaded into the MNs. Confocal microscopy results revealed that rhCOL17p was uniformly distributed within the MNs, and the fluorescence intensity increased with the increase in rhCOL17p concentration (Figure 5E).

### Mechanical strength, skin penetration and sustained release of rhCOL17p from rhCOL17p-MN patches

**Figure 6.**
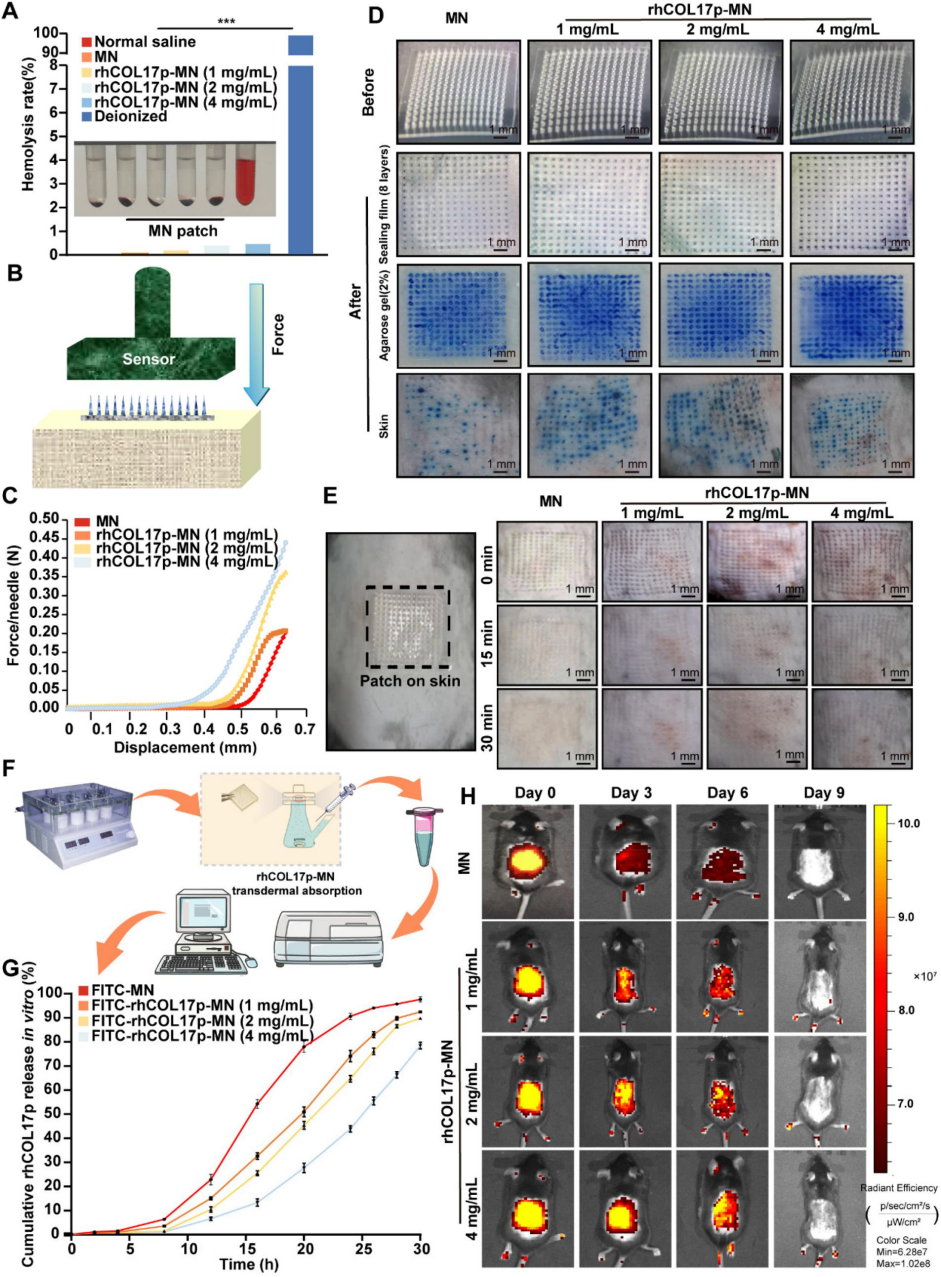
*In vivo* penetration, safety and release profile of rhCOL17p-MN patch. (**A**) Hemolysis activity rhCOL17p-MN patch at different concentrations. (**B**) Schematic diagram of microneedle mechanics under a dynamic universal testing machine. (**C**) Relationship between displacement and pressure curves of microneedles. (**D**) Performance evaluation of rhCOL17p-MN for skin penetration. (**E**) Evaluation of skin micropore healing after penetration by rhCOL17p-MN. (**F**) Transdermal absorption device. (**G**) Quantitative analysis of transdermal absorption of rhCOL17p-MN in tissues. (**H**) Representative fluorescence images of mice skin before and after (i.e. Days 0, 3, 6 and 9) insertion of an FITC-rhCOL17p-MN patch *in vivo*. *P < 0.05, **P < 0.01, ***P < 0.001 and ****P < 0.0001.

A hemolysis test was conducted to evaluate the potential toxic effects of rhCOL17p-loaded dissolving MN (DMN) patches *in vivo*. The test results demonstrated that the hemolysis rates of the MN patches loaded with rhCOL17p at concentrations of 1, 2 and 4 mg/mL were 0.233%, 0.440% and 0.505% (Figure 6A), respectively. These values indicated that the rhCOL17p-MN patches at the tested concentrations (i.e. 1, 2 and 4 mg/mL) exhibit no toxicity.

The mechanical properties of the rhCOL17p-loaded MN patches were assessed using a stress–strain gauge under dynamic force (Figure 6B). The pressure–displacement diagrams revealed that the rhCOL17p-MNs (i.e. 1, 2 and 4 mg/mL) could withstand a force of ≥0.2 N/needle under pressure, surpassing the force required for human skin implantation (≈ 0.1 N/needle). The maximum fracture force was 0.45 N/needle at a concentration of 4 mg/ml (Figure 6C). These findings suggested that the fabricated MNs possess sufficient mechanical strength for skin insertion.

To assess the insertion ability of the MN patches (CM-MNs), patches containing methylene blue such as MN and rhCOL17p-MN (i.e. 1 mg/mL, 2 mg/mL, 4 mg/mL) were inserted into the skin of mice, leaving a series of complete blue micropores on the skin (Figure 6D). This indicated that the MN patches can easily penetrate the stratum corneum, achieving a skin puncture rate of 96%. A 15 × 15 array of optical micropores was visible at the administration site without any additional skin damage. Within 30 min after removing the substrate, the micropores gradually became invisible and completely disappeared (Figure 6E), signifying that the MN patches have excellent skin-implantation ability. Furthermore, blank HA/pullulan MNs dissolved completely in PBS within 3 min (Supplementary Figure S2), indicating that the MNs can rapidly dissolve after insertion, thereby avoiding the retention of solid foreign bodies in the skin and reducing the risk of inflammation, irritation or foreign body reactions.

To investigate the sustained release of rhCOL17p from the rhCOL17p-MN patches and better simulate the degradation and drug-release kinetics *in vivo*, the FITC-rhCOL17p-MN patch was inserted into pig skin to release FITC-rhCOL17p. The changes in FITC during the release process were observed (Figure 6F). As the MN patch gradually dissolved completely, ∼90% of FITC-rhCOL17p was released from the patch within 28 h (Figure 6G).

To evaluate the *in vivo* release and degradation of rhCOL17p from the patches, FITC was added as a tracer. FITC-rhCOL17p-MN patches with different concentrations of rhCOL17p (i.e. 0, 1, 2 and 4 mg/mL) were prepared and inserted into the skin of mice. Fluorescence imaging using an *in vivo* imaging system confirmed the successful insertion of the MN patches into the skin and their uniform distribution on the backs of the mice through the fluorescent dye FITC. As the tracer dye diffused into the mice skin, the fluorescence intensity gradually faded. Compared with the MN group, the FITC-rhCOL17p-MN patch maintained a detectable fluorescence signal in mice for up to 6 days (Figure 6H), indicating that the rhCOL17p-MN patches can maintain the stability of rhCOL17p *in vivo*.

### Evaluation of hair regeneration efficacy of the rhCOL17p-MN patch in AGA mice

**Figure 7.**
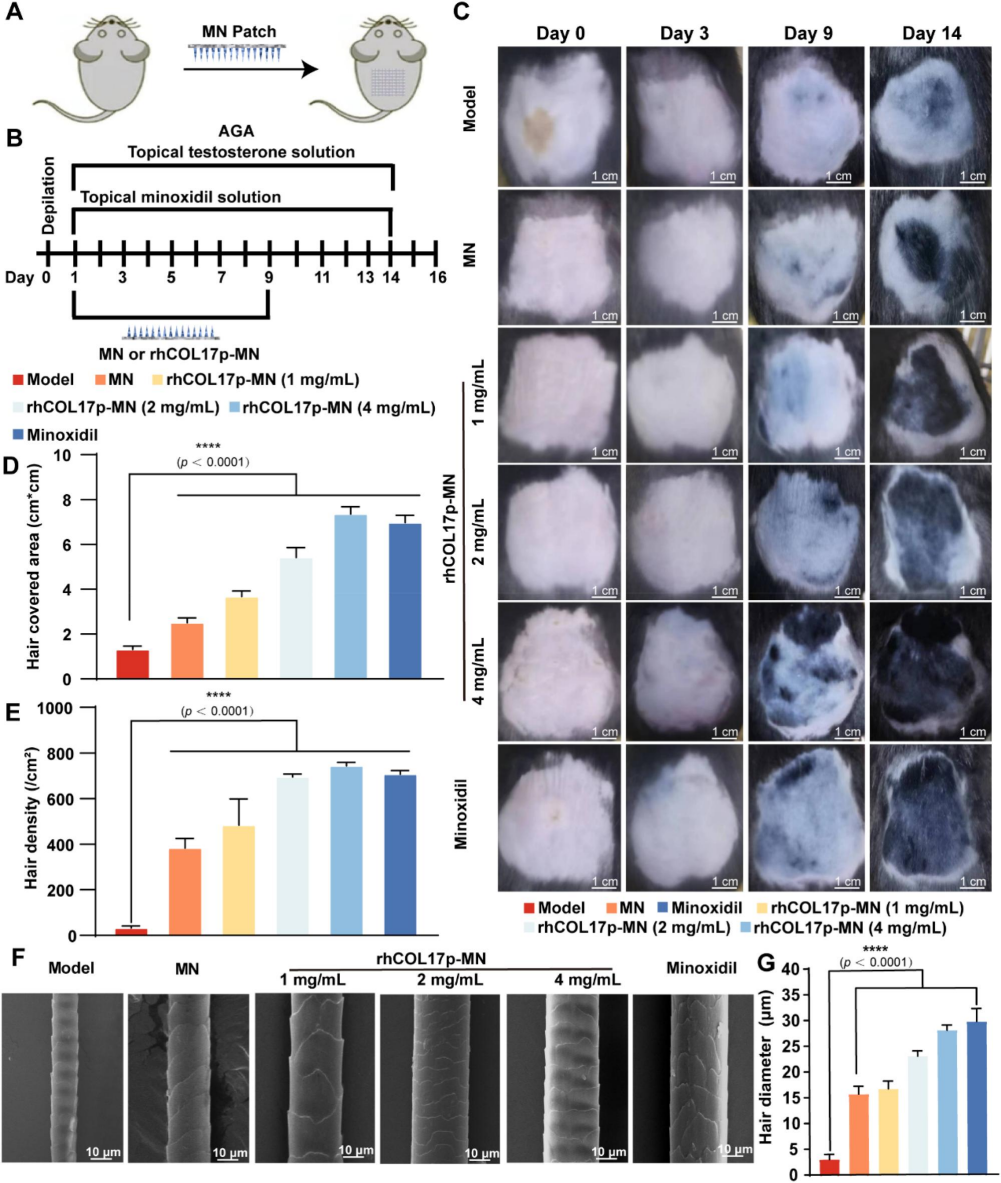
Evaluation of hair regrowth in an AGA-like mouse model. (**A**) Schematic diagram of mice treated with rhCOL17p-MN patch. (**B**) Scheme of hair loss therapy protocol in mice model administrated via rhCOL17p-MN patch and minoxidil. (**C**) Representative photographs of mice treated with model, MN, rhCOL17p-MN patch and minoxidil. (**D**) Quantification of hair regrowth area in AGA mice in each group on Day 14. (**E**) Quantification of the regenerated hair density in AGA mice in each group on Day 14. (**F**) Representative scanning electron microscopy images of regenerated hair in different groups 14 days after depilation. (**G**) Diameter of the regenerated hair in AGA mice in each group on Day 14. Scale bar = 10 μm. Each bar represents the mean±SD (*n* = 5). *P < 0.05, **P < 0.01, ***P < 0.001 and ****P < 0.0001.

To evaluate the hair regeneration potential of rhCOL17p-MN patch, we established an AGA mice model using C57BL/6 male mice, which were randomly allocated into six experimental groups (Figure 7A and B): Model group (topical testosterone only), MN group, rhCOL17p-MN groups (i.e. 1, 2 and 4 mg/mL rhCOL17p loading) and 5% minoxidil positive control group.

The MN and rhCOL17p-MN groups received transdermal patch applications on different times (i.e. Days 1, 3, 5, 7 and 9), while the minoxidil group was administered daily for 9 days. By Day 9, visible hair regeneration was evident in all rhCOL17p-MN groups (i.e. 1, 2 and 4 mg/mL) and the minoxidil group, whereas the model groups exhibited only pigmented hair follicles without shaft emergence (Figure 7C). Importantly, the rhCOL17p-MN groups demonstrated significantly enhanced hair regrowth compared to the MN group. At Day 14, the model groups maintained sparse hair coverage (13.63 ± 0.61)%, while the rhCOL17p-MN groups (all concentrations) and the minoxidil group exhibited dense hair regeneration (Supplementary Figure S1). The 4-mg/mL rhCOL17p group achieved dense hair regrowth that covered 97% of the treated area in the testosterone-exposed mouse model, showing statistical equivalence to daily minoxidil in covered area (7.84 ± 0.37% vs 6.36 ± 1.16%) (Figure 7D), hair density (738.4 ± 8.31 vs 705.6 ± 14.21 hairs/cm^2^) (Figure 7E) and diameter (28.00 ± 0.82 vs 29.67 ± 2.05 μm) (Figure 7F and G). Minor variability in hair diameter between the 2 and 1 mg/mL rhCOL17p-MN groups reflects natural biological heterogeneity and transient transitional hairs during regeneration, without affecting the overall trend of terminal hair thickening compared to the model group. These findings demonstrated that sustained delivery of rhCOL17p dissolving MNs achieves therapeutic efficacy comparable to the minoxidil, highlighting its potential as an alternative treatment for AGA.

### rhCOL17p-MN patch promoted hair follicle regeneration of AGA mice

**Figure 8.**
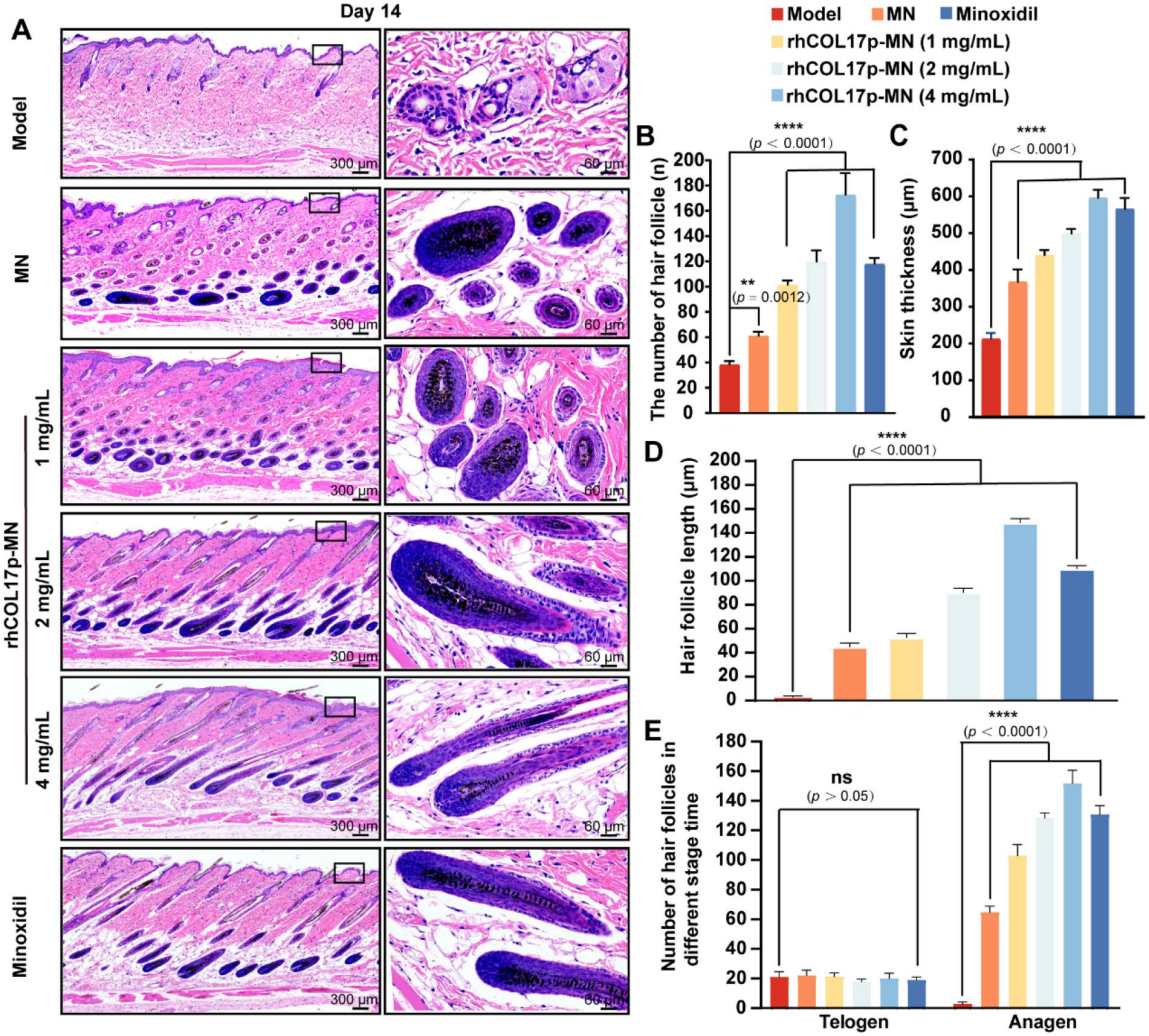
rhCOL17p-MN Patch promoted hair follicle regeneration. (**A**) H&E staining of skin tissue at the treatment site in different groups on Day 14. Scale bars: 300 and 60 μm. (**B**) Quantification of hair follicle number in each group on Day 14. (**C**) Quantification of dermis thickness in each group on Day 14. (**D**) Quantification of hair follicle length in each group on Day 14. (**E**) Quantification of hair follicle number in two phases. Each bar represents the mean±SD (*n* = 5).*P < 0.05, **P < 0.01, ***P < 0.001, ****P < 0.0001 and ns: *P *> 0.05.

Changes in hair follicle length and skin thickness, which are closely associated with the hair cycle, typically increase during the hair growth phase [41]. We analyzed hair follicle numbers through tissue section. After 14 days of treatment, histological analysis revealed that the model group exhibited reduced hair follicle numbers, increased telogen-phase follicles, follicle shrinkage and thinning of the subcutaneous fat layer (Figure 8A). In contrast, the rhCOL17p-MN groups (1, 2 and 4 mg/mL) showed a dose-dependent increase in hair follicle numbers, with the 4 mg/ml group demonstrating the highest count, significantly surpassing the model group (Figure 8B). Skin thickness measurements on Day 14 further supported these findings. The model group had the thinnest skin, while the MN group showed a minor increase. The rhCOL17p-MN groups and the Minoxidil group exhibited significantly thicker skin, with the 4 mg/mL group achieving the greatest thickness, significantly differing from the model group (Figure 8C). Hair follicle length was also evaluated. The model group had shorter follicles, while the MN group showed a slight increase. The rhCOL17p-MN groups and the Minoxidil group demonstrated longer follicles, with the 4 mg/mL group exhibiting the longest, significantly differing from the model group (Figure 8D). Additionally, the distribution of hair follicles in telogen and anagen phases was assessed. The model group had predominantly telogen-phase follicles, while the MN group showed a slight increase in anagen-phase follicles. The rhCOL17p-MN groups and the Minoxidil group exhibited a more pronounced increase in anagen-phase follicles, with the 4 mg/ml group showing the highest number (Figure 8E).

In summary, the rhCOL17p-MN formulation containing 4 mg/ml rhCOL17p demonstrated significantly promoted hair follicle regeneration, increased skin thickness, enhanced follicle length and facilitated the transition from telogen to anagen phase.

### The rhCOL17p-MN patch accelerates hair follicle transition and promotes hair growth by enhancing cell proliferation, and angiogenesis

**Figure 9.**
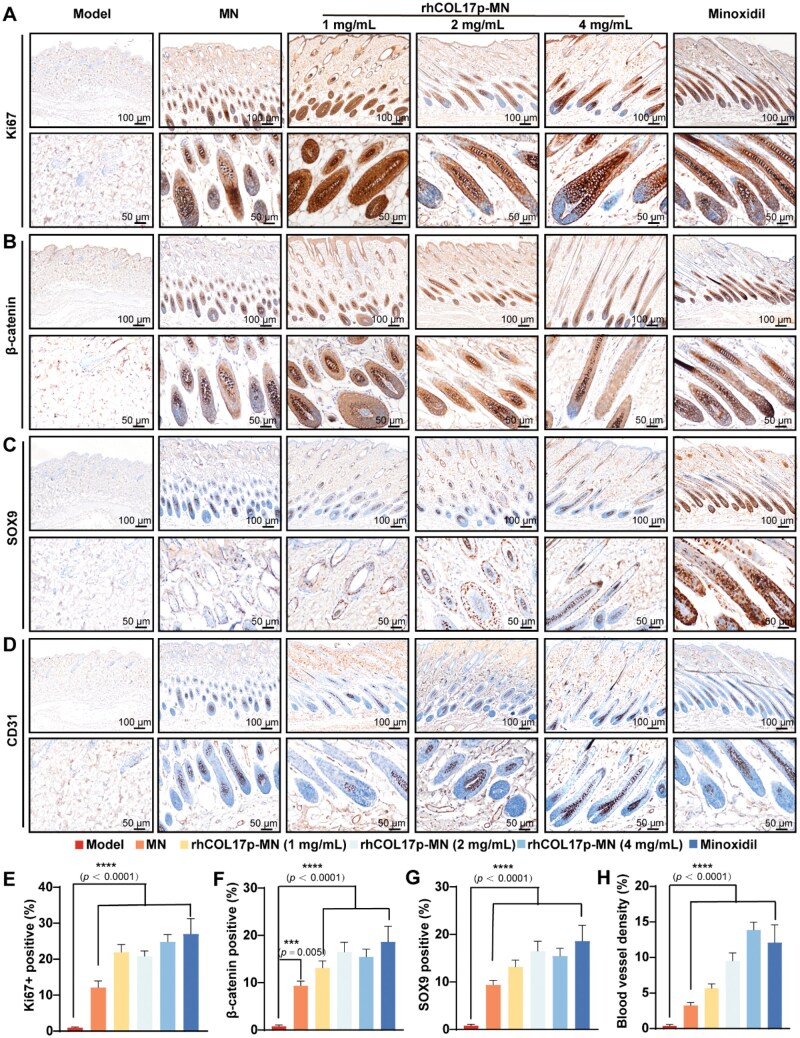
The rhCOL17p-MN patch accelerates hair follicle transition and promotes hair growth by enhancing cell proliferation, and angiogenesis. Immunohistochemical staining of skin sections for (**A**) Ki67, (**B**) β-catenin, (**C**) SOX9 and (**D**) CD31 in groups: Model, MN, rhCOL17p-MN (1/2/4 mg/mL), minoxidil. Shown at ×100 (scale bar = 100 μm) and ×200 (scale bar = 50 μm). markers reflect proliferation (Ki67), hair follicle development (β-catenin, SOX9) and vascular density (CD31). Quantification of (**E**) Ki67⁺, (**F**) β-catenin⁺, (**G**) SOX9⁺ cell percentages and (**H**) CD31⁺ blood vessel density across groups. Each bar represents the mean±SD (*n* = 5). *P < 0.05, **P < 0.01, ***P < 0.001 and ****P < 0.0001.

Hair follicle growth is closely linked to cell proliferation, prompting us to evaluate key markers, Ki67, SOX9, CD31 and β-catenin, during hair regeneration. The rhCOL17p-MN and minoxidil groups exhibited significantly higher Ki67^+^cell proportions compared to the AGA model and blank MN groups (Figure 9A and E), demonstrating their comparable efficacy in promoting hair follicle stromal cell proliferation. Additionally, elevated β-catenin expression in the rhCOL17p-MN and minoxidil groups (Figure 9B and F) further indicated activation of β-catenin signaling, potentially driving hair follicle morphogenesis. Moreover, the rhCOL17p-MN group had a greater number of SOX9^+^ HFSCs than the model group (Figure 9C and G), confirming its ability to promote stem cell differentiation. The rhCOL17p-MN group showed the highest CD31^+^ vessel density (Figure 9D and H), suggesting enhanced angiogenesis to improve the hair follicle microenvironment.

Collectively, these findings demonstrated that the rhCOL17p-MN patch accelerates the transition of hair follicles from telogen to anagen by enhancing proliferation, angiogenesis and β-catenin signaling, effectively promoting hair growth.

### Safety evaluation of rhCOL17p-MN *in vivo*

To evaluate the safety and biocompatibility of rhCOL17p-MN, healthy C57BL/6J male mice were treated with the rhCOL17p-MN for 28 days. After treatment, anesthetized mice were euthanized, and visceral tissues (heart, spleen, liver, lungs and kidneys) were fixed in 4% paraformaldehyde, paraffin-embedded and sectioned for H&E staining. Blood samples were collected for hemolysis testing and to measure liver (ALT, AST) and kidney (Cre, urea, UA) function markers.

**Figure 10.**
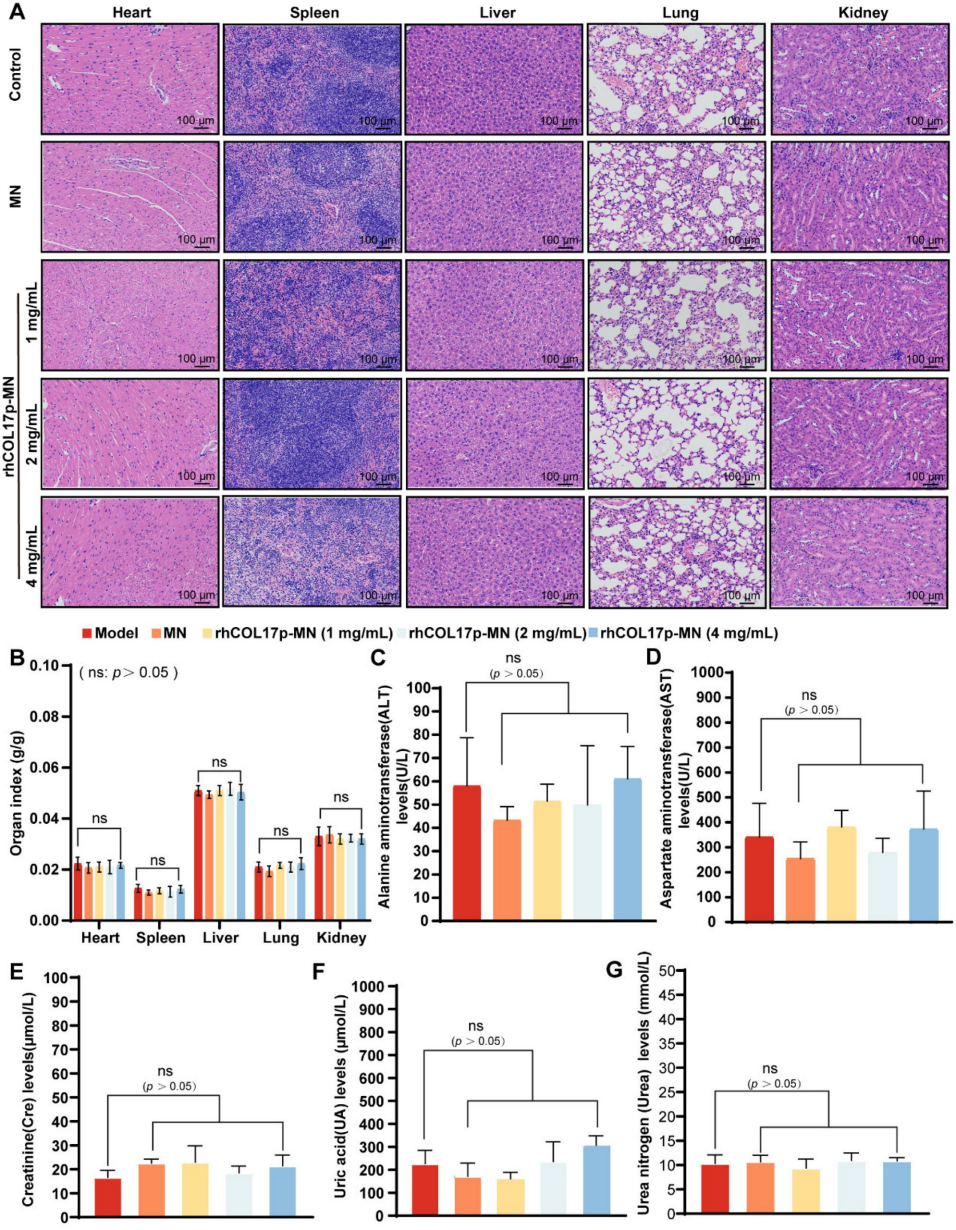
Safety analysis of rhCOL17p-MN patch in mice *vivo* on Day 28. (**A**) H&E staining results of major organs (heart, liver, spleen, lung, kidney) in mice after different treatments. (**B**) Organ index (g/g) of mice after treatment with rhCOL17p-MN patch at different concentrations. (**C–G**) Blood biochemical analysis, such as liver function (ALT, AST), renal function (cre, UA, urea) after application of rhCOL17p-MN patch at different concentrations. Each bar represents the mean±SD (*n* = 5). *P < 0.05, **P < 0.01, ***P < 0.001, ****P < 0.0001 and ns: *P *> 0.05.

H&E staining revealed no morphological differences in visceral tissues between the normal and MN-treated groups (Figure 10A and B), indicating no tissue damage. The hemolysis test, used to assess potential toxicity, demonstrated the safety of rhCOL17p-MN. Additionally, liver and kidney function markers showed no significant differences between the MN-treated and normal groups (Figure 10C–G), confirming good tolerance and no significant inflammation or tissue damage. These results highlight the excellent biocompatibility and safety of rhCOL17p-MN.

## Discussion

In this study, the testosterone-induced AGA-like mouse model revealed a significant downregulation of COL17 in the treatment group compared to control group which correlated with hair follicle shrinkage, decreased proliferative activity (Ki67 downregulation) and reduced angiogenesis (CD31 downregulation). These findings align with the established role of COL17 in maintaining basement membrane integrity [19] and HFSC function [42]. Notably, the concurrent downregulation of β-catenin, a key component of the Wnt signaling pathway [43], suggested that COL17 may regulate the transition of hair follicles from the anagen to telogen phase through β-catenin-mediated signaling. This mechanism highlights COL17 as a critical link between androgen exposure and hair follicle degeneration, providing a potential therapeutic target for AGA. To dissect COL17-independent pathways, we will next employ finasteride co-treatment, inducible *Col17a1* epidermal knock-out mice and human scalp explants from genetically confirmed AGA patients.

To validate the functional relevance of COL17 in hair regeneration, we further investigated the effects of its recombinant functional fragment (rhCOL17p) using *in vitro* assays. Results showed that rhCOL17p dose-dependently promoted hDPCs adhesion, migration and proliferation in a dose-dependent manner. At 25.00 μg/mL, cell migration rates increased by approximately (44.46 ± 4.30)%, with maximal adhesion efficacy observed at 12.50 μg/mL. These effects are likely mediated by the dual role of COL17 as a hemidesmosomal component: its N-terminal domain binds to integrin *α*6*β*4 to facilitate cell-matrix adhesion, while its C-terminal domain activates the FAK-PI3K pathway to promote cytoskeletal rearrangement [44]. The lack of a clear dose-dependent trend in proliferation may reflect receptor saturation, indicating a threshold for optimal cellular response [45]. These findings support COL17’s dual role as both a structural scaffold and a signaling modulator, critical for reconstructing the hair follicle microenvironment and restoring cellular functions essential for hair regeneration [46]. These *in vitro* findings underscore the potential of rhCOL17p to enhance hair follicle-related cellular functions, laying the groundwork for its therapeutic application.

The transdermal route (such as nanogels [47], dissolving MNs [48], nanoparticle-based MN patch [49, 50], buccal film holds [51], silver nanoparticle-loaded MN patch [52]) is an important delivery system for the delivery of both systemic and local effect [53]. Among these, HA dissolving MNs offer rapid skin dissolution, high drug-loading capacity and proven biocompatibility, positioning them as a promising carrier for macromolecule delivery. To overcome the challenge of poor transdermal penetration of macromolecules like rhCOL17p (∼45 kDa), the rhCOL17p-MN patch was developed, representing a significant advancement in drug delivery. The rhCOL17p-MN delivery system offers several technical advantages and translational potential. Its 15 × 15 MN array (750 μm height) achieves 96% skin penetration, enabling sustained release of 90% rhCOL17p within 28 h, which is superior to traditional topical formulations [54]. The patch’s safety profile is supported by hemolysis rates <0.5% and the absence of significant inflammation in histological analyses, confirming its biocompatibility [36]. Furthermore, the 4-mg/mL rhCOL17p concentration emerged as optimal, balancing follicle regeneration metrics and minimizing potential side effects. Meanwhile, we took into account that the mouse hair cycle consists of anagen (growth, ∼2–3 weeks), catagen (regression) and telogen (resting). To optimize dosing frequency for anagen coverage, we assessed rhCOL17p’s release and persistence. *In vitro* Franz-cell assays showed rhCOL17p fully released from MNs within ∼30 h, while *in vivo* tracking revealed FITC-labeled rhCOL17p remained detectable in the dermis for 6 days post-application. Based on this, we dosed mice every 2 days (Days 1, 3, 5, 7, 9) to maintain effective levels over 14 days. Visible hair shafts appeared by Day 9, peaking on Day 14, aligning with the transition from telogen (∼2–3 days) to anagen (∼10–12 days). This confirms rhCOL17p’s release matches the follicle growth ‘critical window’, while subsequent growth relies on intrinsic cycle regulation. Compared to daily minoxidil applications, the patch’s once-every-2-3-days dosing regimen enhances patient compliance, making it a promising alternative for hair loss treatment. This administration frequency avoids potential concentration fluctuations that may occur with single-dose administration, while dynamically supplementing the drug to ensure coverage of the critical stages of hair follicle regeneration, thereby providing an experimental basis for the optimization of administration regimens in clinical translation.

The *in vivo* efficacy of the rhCOL17p-MN patch was further validated in the AGA-like mouse model. The 4-mg/ml rhCOL17p-MN treatment group achieved ∼97% mean hair coverage by Day 14, demonstrating comparable effectiveness to the 5% minoxidil positive control group. Histological analysis revealed increased hair follicle density, longer follicle length and a higher proportion of anagen-phase follicles, accompanied by upregulated expression of Ki67, β-catenin and SOX9. The therapeutic mechanism of the patch involves multiple pathways: enhanced angiogenesis, as evidenced by increased CD31^+^ vascular density, improves nutrient supply to hair follicles [55, 56]; activation of the Wnt signaling pathway through β-catenin nuclear translocation promotes HFSCs proliferation [57] and elevated SOX9^+^ cell numbers suggest that rhCOL17p delays stem cell exhaustion, thereby prolonging regenerative capacity [58]. These results collectively demonstrate the patch’s ability to restore hair follicle function and promote hair regrowth.

Despite these advancements, the study has notable limitations. The use of C57BL/6 mice, which have a short hair cycle, necessitates validation in long-cycle models (e.g. DBA/2 mice) or humanized follicle transplant models. Additionally, interactions between COL17 and other collagens (e.g. COL4, COL7) remain uninvestigated, and the role of COL17 degradation products (e.g. NC1 domain) is unclear. In addition, whether androgens transcriptionally repress COL17, or whether COL17 influences androgen receptor signaling, remains unknown and is under investigation in our laboratory. Future research should focus on developing combination therapies, such as dual-layer MNs co-delivering rhCOL17p and low-dose AR inhibitors, engineering pH-responsive MNs for targeted rhCOL17p release in the acidic follicular microenvironment and assessing long-term safety and pharmacokinetics in porcine skin models. Furthermore, future work may employ subcellular fractionation or immunofluorescence co-localization to directly verify β-catenin nuclear translocation, further refining the mechanistic understanding of COL17’s role in Wnt signaling regulation.

Moreover, while the present study provides comprehensive acute safety data demonstrating the initial biocompatibility of the rhCOL17p-MN patch, we acknowledge that long-term safety evaluation represents a critical next step. In future studies, we propose conducting extended investigations, including 12- to 24-week repeat-dose protocols in mice and preliminary mini-pig implantation trials. These studies will systematically evaluate chronic toxicity, immunogenicity and systemic exposure, ensuring a thorough safety profile before advancing to clinical considerations.

These findings provide proof-of-concept in a testosterone-driven AGA-like mouse model, demonstrating that MN-delivered rhCOL17p promotes hair regeneration by activating stem cells, enhancing angiogenesis. While further validation in larger-animal models and clinical studies is needed, the rhCOL17p-MN patch represents a safe, effective and promising alternative for hair loss therapy.

## Conclusion

This study identified COL17 downregulation as a key mechanism in AGA, linked to abnormal follicle morphology, reduced proliferation and impaired angiogenesis. We then developed rhCOL17p and delivered it via a HA-based dissolving MN patch. *In vivo*, the 4-mg/ml rhCOL17p-MN restored 97% hair coverage by Day 14, comparable to 5% minoxidil, with increased follicle density, anagen-phase transition and CD31^+^ vascularization. Histological analysis revealed upregulated β-catenin and SOX9, indicating stem cell and proliferative pathway activation. The rhCOL17p-MN also demonstrated excellent safety (hemolysis <0.5%) and mechanical stability (≥0.2 N/needle). These findings establish COL17 as a critical target in AGA and highlight rhCOL17p-MN as a promising preclinical evidence for further investigation.

## Supplementary Material

rbaf104_Supplementary_Data

## Data Availability

Data will be made available on request.
