# Supplementary material for: Engineered collagen XVII-loaded dissolving microneedle patch for promoting hair regrowth in androgenic alopecia
Source: Regen Biomater. 2025 Nov 9;12:rbaf104. doi: 10.1093/rb/rbaf104 (PMC12688376; doi:10.1093/rb/rbaf104)
Supplement: rbaf104_Supplementary_Data [file rbaf104_supplementary_data.docx]

**Supplementary Figures and Tables**

**Tab. S1. Physicochemical characteristics of hCOL7 and hCOL17p**

| Physical and chemical indicators | hCOL17 | rhCOL17p |
| --- | --- | --- |
| Formula | C_6533_H_10288_N_1896_O_2109_S_40_ | C_1434_H_2196_N_380_O_464_S_5_ |
| Theoretical molecular weight | 150419.39 | 32586.05 |
| Theoretical pI | 8.89 | 4.54 |
| Total number of atoms | 20866 | 4514 |
| Ext.coefficient (280nm)L·mol^-^¹·cm^-^¹‌ | 109015 | 0.763 |
| Estimated half-life (h) | 30 hours (mammalian reticulocytes, *in* *vitro*); > 20 hours (yeast, *in* *vivo*); > 10 hours (Escherichia coli, *in vivo*). | |
| Instability index | 45.25 | 53.24 |
| Aliphatic index | 55.47 | 62.76 |
| Grand average of hydropathicity (GRAVY) | - 0.573 | - 0.413 |

**Tab. S2. Functional domain prediction analysis of hCOL17p**

| Name | Start | End | E-value | Function |
| --- | --- | --- | --- | --- |
| Pfam:Collagen | 1 | 58 | 0.00046 | threshold |
| MAGE_N | 29 | 101 | 150000 | - |
| Pfam:Collagen | 64 | 128 | 0.00075 | threshold |
| LNS2 | 120 | 323 | 101000 | - |
| Pfam:LisH | 127 | 139 | 32 | The LisH domain exists in Sif2, which is a component of the Set3 complex responsible for inhibiting meiotic division genes. Research has shown that the LisH domain helps mediate interactions with Set3 complex components. |
| Pfam:Collagen | 146 | 168 | 0.53 | threshold |
| Pfam:LisH | 173 | 194 | 31 | Participate in microtubule dynamics, cell migration, nuclear movement, and chromosome separation within eukaryotic cells. Associated with other domains such as WD-40, SPRY, Kelch, AAA ATPase, RasGEF, or HEAT. |
| Efhand Cainsen | 176 | 251 | 162000 | - |
| SMI1_KNR4 | 196 | 282 | 116000 | - |
| ARM | 198 | 255 | 1980 | - |
| Pfam:Collagen | 219 | 236 | 3.2 | threshold |
| Pfam:Collagen | 257 | 309 | 0.25 | threshold |


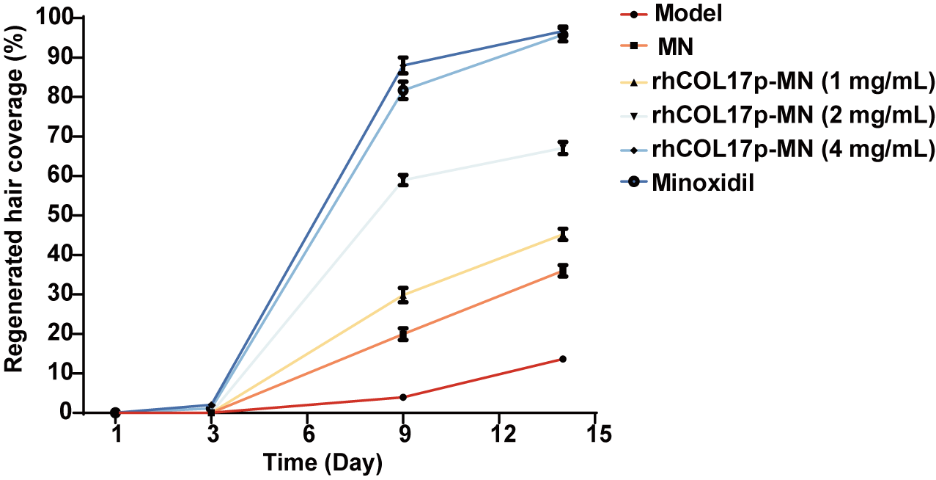


**Fig. S1. Analysis of hair coverage in each group of mice.**

**
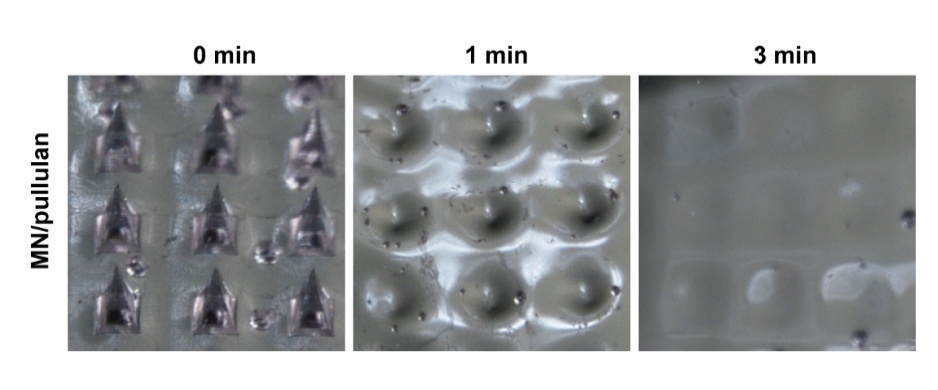
**

**Fig. S2. *In vitro* dissolution time of blank (HA/pullulan) microneedles.**
